# Whistle-blowing and duty of candour in the National Health Service: a ‘history and policy’ case study of the 1960s and 2010s

**DOI:** 10.1177/0141076816658787

**Published:** 2016-08-17

**Authors:** Claire Hilton

**Affiliations:** Consultant old age psychiatrist, Brent Memory Service, London NW 9 0PS, UK

## Introduction

The Mid Staffordshire Public Inquiry (Francis Report) in 2013^1^ identified a catalogue of preventable harm, including deaths, of patients in a NHS general hospital. This paper uses historical sources from the 1960s to help explore two interacting themes raised in the Report, the duty of candour and whistle-blowing, with the aim of informing current debate. Whistle-blowing and duty of candour deserve consideration particularly during periods of austerity, as the NHS is experiencing now, since under-resourced clinical teams struggling to cope are more likely to cut corners compromise care and be defensive if criticised.

## Duty of candour and whistle-blowing

Since 2014, the NHS has had to comply with a statutory duty of candour.^2^ This means that staff must be honest with patients and their families when an untoward incident, for which they are responsible, causes physical or ‘prolonged psychological’ harm or death. Whistle-blowing is when a staff member exposes information or activity that is deemed illegal, unethical or otherwise likely to cause harm, and for which intervention may reduce future damage. Blowing the whistle is not an easy option. Most people like to be accepted by their colleagues, tend to conform to group practices and do not challenge authority. Hence, metaphorically stepping outside one’s own work group to criticise it, is psychologically challenging.^3^ The Francis Report identified staff who were scared to speak out, and when they did, senior staff were hostile towards them and defensive of existing practices.^4^ This fits with the old adage that staff should ‘give in or get out’: giving in and accepting the *status quo* is easier than getting out.

When Minister of Heath Kenneth Robinson was asked in 1967 if he would protect whistle-blowers from victimisation, he said ‘Yes, certainly’, but he gave no clue as to how he would achieve this.^5^ The Ministry of Health nebulously advised the Regional Hospital Boards, which managed the hospitals, to try to ‘dispel such apprehensions’ about whistle-blowers being victimised.^6^ Two years later, the *Nursing Mirror* commented that when nurses speak out, ‘the painful truth is that, invariably, their own discredit is the only result of their efforts.’^7^ Fifty years on, new NHS guidance encourages staff to speak out and reassures them: ‘We will look into what you say and you will always have access to the support you need.’ The guidance also states that it provides a ‘vision for raising concerns’,^8^ but if Health Secretary Jeremy Hunt genuinely wants to abolish a NHS ‘cover-up culture’,^9^ action needs to be prompt rather than an optimistic vision.

## Barbara Robb and her campaign to improve care, 1965–1974

In 1965, Barbara Robb visited a 73-year-old acquaintance, an inpatient on a long-stay psychiatric ward at Friern Hospital, London. Robb was appalled by standards of care, including: slapping patients; dreadful food; bed time at 7 p.m.; lack of dentures, spectacles and hearing aids, which discouraged independence; and lack of activities, personal clothes and privacy, which undermined dignity and self-esteem.

Robb began her campaign to improve care after senior NHS officials ignored her complaints and she realised that similar conditions were widespread. She established Aid for the Elderly in Government Institutions, a small, elite, pressure group. Aid for the Elderly in Government Institutions’ expert advisors included Russell Barton, a pioneering psychiatrist, and Brian Abel-Smith, government advisor on social policy and professor at the London School of Economics. In 1967, Robb compiled the book *Sans Everything: a case to answer.*^10^ It comprised eye-witness reports of inhumane care of dependent and frail older people in seven NHS hospitals, plus commentary and proposals by her organisation's advisors about making improvements. Proposals included creating dedicated psychiatric services for older people, developing effective NHS complaints procedures, appointing an independent health service ombudsman and establishing a hospitals’ inspectorate.

Aid for the Elderly in Government Institutions built strong links with the press, which raised public awareness and exerted pressure on the Ministry. Regional Hospital Boards established committees of inquiry into the *Sans Everything* allegations. The committees based their judgements on stereotypical, outdated clinical knowledge and negative views of older people and mental illness, and on deep faith in the excellence of the NHS. The committees used leading questions and discredited the author-witnesses (e.g. over-sensitive, insufficiently objective, too sentimental) rather than evaluating their evidence. These biased processes enabled them to conclude that most of the allegations were false.^11^

The press and public supported Robb, but NHS authorities, including Robinson and the Regional Hospital Boards, condemned her as irresponsible.^12^ Robb had extraordinary determination, personal resilience and energy to persist in the face of public humiliation. Her work triggered similar allegations in other hospitals. Inquiries at these hospitals upheld almost all allegations, which vindicated her. Their outcomes, together with Robb’s ongoing campaign, contributed to NHS policy changes to raise and monitor standards, linked to proposals made in *Sans Everything*.

## Obeying orders

In the 1960s, nurses were expected to automatically obey instructions from their superiors. Hierarchical, authoritarian top-down management discouraged front-line staff from asking questions. Staff could be disciplined for querying instructions, even if attempting to improve care.^13^ Demeaning practices which passed unchallenged were perpetuated. Today, unquestioning obedience to orders is associated with rigid clinical protocols and ‘care pathways’ which are carefully monitored to ensure task-focused, rather than patient-focused, implementation. Pathways, created by experts and assumed to be best for patients, may not be.^14^ Their rigidity gives staff little scope for questioning, innovating or tailoring care to an individual patient’s needs. Andy Burnham, a former Health Minister, said ‘the NHS is not good at giving its front-line staff a sense of empowerment. People with good ideas do not feel that they can easily put them into action.’^15^ Dis-empowerment will also discourage staff from the more challenging task of raising concerns.

## The ‘best in the world’ rhetoric: then and now

Robb found an impenetrable wall of secrecy, defensiveness and denial of poor practice within the NHS. One Regional Hospital Board chairman wrote to *The Times* that Regional Hospital Boards aimed to ‘guard and protect the interest and care of patients’,^16^ and stated on television, ‘we are on the side of the patient. That is what we are there for.’^17^ However, he ignored poor standards which were reality for many patients, including at Friern. This linked to government reassurance that the NHS was the ‘best health service in the world’,^18^ a widely believed perspective, but one unsupported by data. Comparative health outcomes were in their infancy.^19^ Abel-Smith did not have, but wanted to obtain, comparative economic data.^20^ ‘Best’ was a political rather than medical or economic endpoint. Robinson stated: ‘I am absolutely sure, that the care of our old people in our geriatric and psychiatric hospitals is as good as anything in the world.’^21^ Similar to the response when resources are limited, that one is ‘doing one’s best under the circumstances’,^22^ it was a relative rather than absolute ‘best’. These expressions acknowledged deficits, but engendered complacency towards low standards. They underpinned the tendency to repeatedly congratulate staff for their dedication and high standards of work. Praise is important: it can help maintain morale. However, staff can perceive excessive praise as shallow, and it defends existing standards and detracts from the authorities’ responsibility to encourage staff to improve care and treatment.

One widely quoted study of 11 Western countries in 2014 identified the NHS as top for ‘quality care’, access and efficiency, but almost bottom (10/11) on the criterion ‘healthy lives’, i.e. its clinical effectiveness defined by the outcome of interventions. ‘Healthy lives’ was weighted the same as the other measures, rather than identifying it as an overarching objective.^23^ The Commonwealth Fund, a United States organisation aiming to create equitable healthcare, sponsored the research. This is not suggesting that the research was misconducted, but that political motives might have contributed to defining and interpreting ‘best’. ‘Best in the world’ statements support complacency to criticism. They reinforce practices of ignoring whistle-blowers and misjudging them as unreasonable.

## Whistle-blowers, loyalty and resources

One *Sans Everything* whistle-blower observed staff swearing, hitting and roughly handling elderly patients, and communal bathrooms where 44 elderly patients were disrespectfully bathed in a single morning. When she complained about staff behaviours, she was taken off duties with patients and transferred to cleaning copper pipes in the bathroom. Her colleagues were angry with her, saying that her comments created an unpleasant work atmosphere and that nurses should be loyal and unified. She resigned.

Russell Barton wrote in the foreword to *Sans Everything* of the ‘misplaced loyalty of one staff member to another. […] Victimisation of anyone who is critical, whether justifiably or not, may be automatic.’ One nurse in 1967 received a letter from her matron: ‘I feel that your disloyalty towards your colleagues and the fact that you are not happy with conditions […], leaves me with no alternative but to ask you to accept one week’s notice.’^24^ Archives do not indicate if the underlying reasons for the discontent were remedied. In 2013, the Francis Report noted an ‘unhealthy culture’ of the primacy of ‘loyalty to the organisation’ within the NHS and social care inspectorate, and powerful staff loyalty in the hospital it had assessed.^25^ The etiquette of loyalty to an organisation rather than to patients can affect staff perspectives on speaking out.

In the 1960s, under-resourcing linked to ward overcrowding (possibly twice as many beds as planned) and understaffing (e.g. one trained and three untrained staff for 84 ‘ambulant geriatric’ patients).^26^ In 2016, overcrowding has transmuted from visible ‘spatial overcrowding’ of beds on a ward, into ‘temporal overcrowding’, with rapid throughput of patients who are discharged to their own homes before they are physically well.^27^ Both sorts of overcrowding/understaffing are associated with a heavy nursing workload which risks compromising care. Today, wards are expected to inform senior hospital managers if staff levels drop below those necessary to maintain safe and humane care. However, this plan has revealed another pitfall, as some hospitals fail to respond to these urgent cries for help^28^: empty-handed outcomes following undertakings by seniors can deter staff from speaking up when faced with other situations which are likely to undermine care.

If sub-standard practices develop, they may become accepted as the norm and pass unnoticed except by new staff or visitors. Most *Sans Everything* whistle-blowers were new to the hospital, idealistic about the well being of their patients, and lacked formal health service related professional qualifications. In other hospitals, *Sans Everything* inspired students to blow the whistle, but their allegations were generally ignored. Unqualified staff, and students who had not yet acquired the views which their professions were *meant* to hold, provided a ‘new pair of eyes’ and enlightened insights into quality of care. Yet these groups were, and are, the least likely to be asked for their views. It might be valuable to routinely seek feedback from them, preferably face to face, if necessary by a member of staff in a different department, taking into account ongoing insecurities about whistle-blowing. Training students and staff in the mechanisms of how to raise concerns is widespread, but they also need to appreciate the art and ethics of doing it. Ethics, beyond the traditional principles of justice, beneficence, non-maleficence and respect for autonomy as they apply to clinical treatment of an individual patient, need to be taught in relationship to health service organisation and management more broadly. This might give all staff a greater understanding of their roles, duties and loyalties and might contribute to shifting NHS culture towards accepting constructive criticism.

## Conclusions

The hospitals about which *Sans Everything* reported have gone, but unsafe, damaging or disrespectful patient care continues. Poor care is connected to ‘people factors’, institutional and personal, some of which today are uncomfortably similar to the 1960s. Under-resourcing may inadvertently encourage inadequate clinical practices. These may be justified as ‘best under the circumstances’. They can take root and propagate if left unchallenged. Unresponsiveness to requests to remedy difficulties, and hostility to whistle-blowers, may exacerbate the situation. Failure to report concerns is amplified in a culture of inflexible monitoring of task-focused clinical pathways and protocols which preclude questioning and innovation.

The duty of candour might contribute to changing the traditional, hostile response to whistle-blowers in the NHS. However, changing institutional culture and behaviours is usually slow. Bottom-up as well as top-down interventions are required, including making the most of a ‘new pair of eyes’ and helping staff understand the ethical basis of responsibility within a health service. Robb wrote that harmful practices in the NHS were ‘an indictment of every one of us who knew these things were happening and did nothing about it.’^29^ Her comment stands today.

## Declarations

### Guarantor

CH

### Provenance

Not commissioned; editorial review

## References

[1] Mid Staffordshire NHS Foundation Trust. Mid Staffordshire NHS Foundation Trust Public Inquiry HC. 947 (Francis Report), London: TSO, 2013.

[2] Health and Social Care Act 2008 (Regulated activities) Regulations 2014. Regulation 20.

[3] Milgram S. Behavioral study of obedience. *J Abnorm Soc Psych*.1963; 67: 371–378; Haney C, Banks C and Zimbardo P. Interpersonal dynamics in a simulated prison. *Int J Crimin Penol* 1973; 1: 69–97. 10.1037/h0040525 14049516

[4] Francis Report. 1.194-1.209.

[5] Mental Hospitals (Conditions), *Hansard* HC Deb 26 June 1967 vol. 749 cc69-70.

[6] Ministry of Health, Letter to RHBs, 28 June 1967, MH.150/350 (TNA, The National Archives, Kew).

[7] Anon. Editorial. *Nursing Mirror*, 24 January 1969.

[8] NHS Improvement and NHS England*. Freedom to Speak Up: Raising Concerns (whistleblowing) Policy for the NHS.* See https://improvement.nhs.uk/resources/freedom-to-speak-up-whistleblowing-policy-for-the-nhs/ (last checked 31 May 2016).

[9] Donnelly L and Sawer P. Hunt: sweeping reforms to end ‘NHS cover up culture’. *Telegraph*, 7 February 2015.

[10] Robb B Sans Everything: A Case to Answer, London: Nelson, 1967.

[11] Ministry of Health. *Findings and Recommendations Following Enquiries into Allegations Concerning the Care of Elderly Patients in Certain Hospitals Cmnd. 3687.* London: HMSO, 1968, p.81.

[12] *Sans Everything* (Reports of Inquiries), *Hansard* HC Deb 09 July 1968 vol. 768 cc213-6.

[13] Dickinson T ‘Curing Queers’: Mental nurses and their patients, 1935–1974, Manchester: Manchester University Press, 2015, pp. 145–148.

[14] Department of Health. *More Care, Less Pathway: A Review of the Liverpool Care Pathway.* See www.gov.uk/government/uploads/system/uploads/attachment_data/file/212450/Liverpool_Care_Pathway.pdf (last checked 31 May 2016).

[15] Francis Report. 20.67.

[16] Hackett M. Old people in mental hospitals. *Times*, 18 November 1965, p.13.

[17] BBC1. *24 Hours*, 28 July 1967, transcript, AEGIS/1/6 (AEGIS Archive, at London School of Economics).

[18] Mental Health Service. Hansard HC Deb 19 March 1965, vol. 708 cc1645-719.

[19] Schieber G Health expenditures in major industrialized countries, 1960-87. Health Care Financ R 1990; 11: 159–167. PMC4193120 10113400

[20] Brian Abel-Smith. Memo, May 1970, 154/3/DH/47/68 (University of Warwick Modern Records Centre).

[21] BBC1. *24 Hours*, 30 June 1967, transcript, AEGIS/1/6.

[22] Martin J, with Evans D. *Hospitals in Trouble.* Oxford: Blackwell, 1984, p.245.

[23] Davis K Stremikis K Squires D Schoen C Mirror, Mirror on the Wall: How the Performance of the US Health Care System Compares Internationally, New York: Commonwealth Fund, 2014.

[24] Ministry of Health, Letter, Matron to Mrs Glynn, 17 August 1967, MH 160/653 (The National Archives).

[25] Francis Report. 6.386, 22.81, 22.86.

[26] Friern Hospital Management Committee agenda papers: General Nursing Council, 7 September 1967, H12/CH/A/14 (London Metropolitan Archives).

[27] Parliamentary and Health Service Ombudsman. *A Report of Investigations into Unsafe Discharge from Hospital.* See www.ombudsman.org.uk/about-us/news-centre/press-releases/2016/vulnerable-patients-and-their-families-suffering-harrowing-ordeals-due-to-poor-hospital-discharge (last checked 31 May 2016).

[28] Unison. Unison’s Staffing Level Survey 2015: Red Alert: Unsafe Staffing Levels Rising, London: Unison, 2015, pp. 7–7.

[29] Robb B. Conditions in mental hospitals. *Times*, 24 June 1968, p.9.

